# Meats and Their Digestibility

**Published:** 1889-11

**Authors:** 


### MEATS AND THEIR DIGESTIBILITY.

According to Payen, without there being anything absolute in those qualities which depend on the particular state of the digestive organs of different individuals or on their idiosyncrasies, we may say in general that meats are more easily digestible the less strong their cohesion and the less their hardness. We might thus establish between them the following order, beginning with the lightest :—Sea and river fish, fowl, game, crustaceans, lamb, veal, beef, mutton, wild boar and pork.

In these categories are generally considered heavy and hard to digest, salmon, eels, geese, ducks and some other water birds, as well as strongly smoked and salted meat.

ON THE TIME REQUIRED FOR THE DIGESTION OF DIFFERENT KINDS OF FOOD.

| | Hours. | | Hours. |
|---|---|---|---|
| Roasted pork | 5.15 | Boiled milk | 2.00 |
| Salt beef (boiled) | 4.15 | Boiled codfish | 2.00 |
| Veal (boiled) | 4.00 | Venison steak | 1.35 |
| Boiled hens | 4.00 | Trout (broiled) | 1.30 |
| Roasted mutton | 3.15 | Tripe | 1.00 |
| Boiled beef | 3.30 | Pig's feet | 1.00 |
| Roasted beef | 3.00 | Eggs (hard boiled) | 3.30 to 5.30 |
| Raw oysters | 2.45 | Eggs (soft boiled) | 3.00 |
| Roasted turkey | 2.30 | | |

The above is taken from Beaumont's "Experiments on digestion." Dalton comments on these observations as follows—"These results would not always be precisely the same for different persons, since there are variations in this respect according to age and temperament. Thus, in most instances, mutton would probably be equally digestible with beef, or perhaps more so ; and milk, which in some persons is easily digested, in others is disposed of with considerable difficulty. But as a general rule the comparative digestibility of different substances is no doubt correctly expressed by the above list."

